# 2-Methyl-*N*′-[1-(2-pyrid­yl)ethyl­idene]benzohydrazide

**DOI:** 10.1107/S1600536810054267

**Published:** 2011-01-08

**Authors:** Chun-Bao Tang

**Affiliations:** a Department of Chemistry, Jiaying University, Meizhou 514015, People’s Republic of China

## Abstract

In the title compound, C_15_H_15_N_3_O, the dihedral angle between the pyridine and benzene rings is 36.3 (2)°. In the crystal, mol­ecules are linked through inter­molecular N—H⋯O hydrogen bonds, forming chains along the *b* axis.

## Related literature

For general background to hydrazones, see: Rasras *et al.* (2010 ▶); Pyta *et al.* (2010 ▶); Angelusiu *et al.* (2010 ▶). For related structures, see: Fun *et al.* (2008 ▶); Singh & Singh (2010 ▶); Ahmad *et al.* (2010 ▶); Tang (2010 ▶). For reference bond-length data, see: Allen *et al.* (1987 ▶).
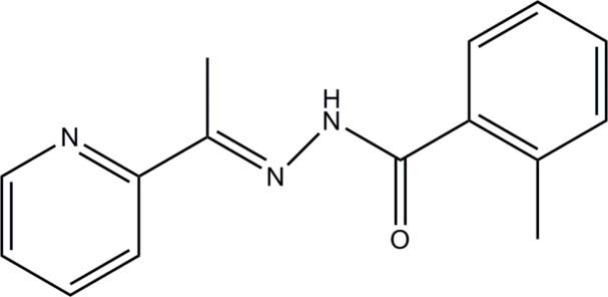


## Experimental

### 

#### Crystal data

C_15_H_15_N_3_O
*M* *_r_* = 253.30
Orthorhombic, 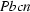

*a* = 19.296 (3) Å
*b* = 8.1417 (18) Å
*c* = 17.294 (3) Å
*V* = 2716.8 (9) Å^3^
*Z* = 8
Mo *K*α radiation
μ = 0.08 mm^−1^
*T* = 298 K
0.20 × 0.20 × 0.18 mm

#### Data collection

Bruker SMART CCD area-detector diffractometer
Absorption correction: multi-scan (*SADABS*; Sheldrick, 1996 ▶) *T*_min_ = 0.984, *T*_max_ = 0.986
13661 measured reflections
2966 independent reflections
1539 reflections with *I* > 2σ(*I*)
*R*_int_ = 0.069

#### Refinement

*R*[*F* ^2^ > 2σ(*F* ^2^)] = 0.056
*wR*(*F* ^2^) = 0.158
*S* = 1.02
2966 reflections
177 parameters
1 restraint
H atoms treated by a mixture of independent and constrained refinement
Δρ_max_ = 0.17 e Å^−3^
Δρ_min_ = −0.17 e Å^−3^

### 

Data collection: *SMART* (Bruker, 2002 ▶); cell refinement: *SAINT* (Bruker, 2002 ▶); data reduction: *SAINT*; program(s) used to solve structure: *SHELXS97* (Sheldrick, 2008 ▶); program(s) used to refine structure: *SHELXL97* (Sheldrick, 2008 ▶); molecular graphics: *SHELXTL* (Sheldrick, 2008 ▶); software used to prepare material for publication: *SHELXTL*.

## Supplementary Material

Crystal structure: contains datablocks global, I. DOI: 10.1107/S1600536810054267/sj5083sup1.cif

Structure factors: contains datablocks I. DOI: 10.1107/S1600536810054267/sj5083Isup2.hkl

Additional supplementary materials:  crystallographic information; 3D view; checkCIF report

## Figures and Tables

**Table 1:** Hydrogen-bond geometry (Å, °)

*D*—H⋯*A*	*D*—H	H⋯*A*	*D*⋯*A*	*D*—H⋯*A*
N1—H1⋯O1^i^	0.91 (1)	2.05 (1)	2.937 (2)	165 (3)

Symmetry code: (i) 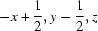.

**Fig. 1.**
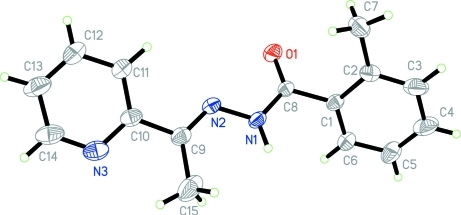
The molecular structure of the compound, showing the atom-numbering scheme. Displacement ellipsoids are drawn at the 30% probability level. Hydrogen atoms are shown as spheres of arbitrary radius.

**Fig. 2.**
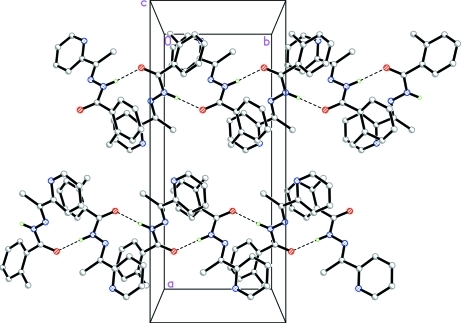
Molecular packing of the title compound, with hydrogen bonds shown as dashed lines.

## supplementary crystallographic information

### Comment

Hydrazone compounds have received much attention in biological and structural chemistry in the last few years (Rasras *et al.*, 2010; Pyta *et al.*, 2010; Angelusiu *et al.*, 2010; Fun *et al.*, 2008; Singh & Singh, 2010; Ahmad *et al.*, 2010). In the present paper, the author reports the crystal structure of the new title hydrazone compound (Fig. 1).

In the title compound, the dihedral angle between the pyridine and the benzene rings is 36.3 (2)°. The torsion angles C1—C8—N1—N2, C8—N1—N2—C9, and N1—N2—C9—C10 are 7.8 (2), 3.6 (2), and 1.5 (2)°, respectively. Bond lengths in the molecules are normal (Allen *et al.*, 1987) and comparable to those in the similar compound the author reported recently (Tang, 2010).

In the crystal structure, molecules are linked through intermolecular N—H···O hydrogen bonds (Table 1), forming chains along the *b* axis (Fig. 2).

### Experimental

2-Acetylpyridine (0.1 mmol, 12.1 mg) and 2-methylbenzohydrazide (0.1 mmol, 15.0 mg) were dissolved in methanol (20 ml). The mixture was stirred at reflux for 10 min to give a clear colourless solution. Colourless block-shaped crystals of the compound were formed by slow evaporation of the solvent over several days.

### Refinement

The amino H atom was located in a difference Fourier map and refined isotropically, with the N—H distances restrained to 0.90 (1) Å [*U*_iso_(H) = 0.08 Å^2^]. Other H atoms were constrained to ideal geometries and refined as riding, with Csp^2^—H = 0.93 Å, and C(methyl)—H = 0.96 Å; *U*_iso_(H) = 1.2*U*_eq_(C) and 1.5*U*_eq_(C_methyl_).

### Crystal data

**Table:**

C_15_H_15_N_3_O	*D*_x_ = 1.239 Mg m^−^^3^
*M**_r_* = 253.30	Mo *K*α radiation, λ = 0.71073 Å
Orthorhombic, *P**b**c**n*	Cell parameters from 1243 reflections
*a* = 19.296 (3) Å	θ = 2.5–24.6°
*b* = 8.1417 (18) Å	µ = 0.08 mm^−^^1^
*c* = 17.294 (3) Å	*T* = 298 K
*V* = 2716.8 (9) Å^3^	Block, colourless
*Z* = 8	0.20 × 0.20 × 0.18 mm
*F*(000) = 1072	

### Data collection

**Table:**

Bruker SMART CCD area-detector diffractometer	2966 independent reflections
Radiation source: fine-focus sealed tube	1539 reflections with *I* > 2σ(*I*)
graphite	*R*_int_ = 0.069
ω scans	θ_max_ = 27.0°, θ_min_ = 2.4°
Absorption correction: multi-scan (*SADABS*; Sheldrick, 1996)	*h* = −24→24
*T*_min_ = 0.984, *T*_max_ = 0.986	*k* = −10→10
13661 measured reflections	*l* = −13→22

### Refinement

**Table:**

Refinement on *F*^2^	Primary atom site location: structure-invariant direct methods
Least-squares matrix: full	Secondary atom site location: difference Fourier map
*R*[*F*^2^ > 2σ(*F*^2^)] = 0.056	Hydrogen site location: inferred from neighbouring sites
*wR*(*F*^2^) = 0.158	H atoms treated by a mixture of independent and constrained refinement
*S* = 1.02	*w* = 1/[σ^2^(*F*_o_^2^) + (0.0493*P*)^2^ + 0.5475*P*] where *P* = (*F*_o_^2^ + 2*F*_c_^2^)/3
2966 reflections	(Δ/σ)_max_ < 0.001
177 parameters	Δρ_max_ = 0.17 e Å^−^^3^
1 restraint	Δρ_min_ = −0.17 e Å^−^^3^

### Special details

**Table:**

Geometry. All esds (except the esd in the dihedral angle between two l.s. planes) are estimated using the full covariance matrix. The cell esds are taken into account individually in the estimation of esds in distances, angles and torsion angles; correlations between esds in cell parameters are only used when they are defined by crystal symmetry. An approximate (isotropic) treatment of cell esds is used for estimating esds involving l.s. planes.
Refinement. Refinement of F^2^ against ALL reflections. The weighted R-factor wR and goodness of fit S are based on F^2^, conventional R-factors R are based on F, with F set to zero for negative F^2^. The threshold expression of F^2^ > 2sigma(F^2^) is used only for calculating R-factors(gt) etc. and is not relevant to the choice of reflections for refinement. R-factors based on F^2^ are statistically about twice as large as those based on F, and R- factors based on ALL data will be even larger.

### Fractional atomic coordinates and isotropic or equivalent isotropic displacement parameters (Å^2^)

**Table:**

	*x*	*y*	*z*	*U*_iso_*/*U*_eq_	
N1	0.26051 (10)	0.9439 (2)	0.05651 (11)	0.0451 (5)	
N2	0.28738 (9)	1.0589 (2)	0.00578 (11)	0.0450 (5)	
N3	0.43437 (11)	1.1437 (2)	−0.10682 (12)	0.0623 (6)	
O1	0.17755 (8)	1.12421 (19)	0.09416 (11)	0.0613 (5)	
C1	0.16962 (11)	0.8519 (3)	0.14164 (13)	0.0423 (5)	
C2	0.10054 (12)	0.8104 (3)	0.12641 (14)	0.0513 (6)	
C3	0.07187 (15)	0.6857 (4)	0.17027 (18)	0.0751 (9)	
H3	0.0266	0.6531	0.1602	0.090*	
C4	0.10778 (18)	0.6085 (4)	0.22804 (19)	0.0832 (10)	
H4	0.0866	0.5263	0.2569	0.100*	
C5	0.17470 (16)	0.6520 (3)	0.24329 (16)	0.0689 (8)	
H5	0.1990	0.6005	0.2829	0.083*	
C6	0.20621 (13)	0.7725 (3)	0.19965 (14)	0.0536 (6)	
H6	0.2521	0.8007	0.2092	0.064*	
C7	0.05886 (13)	0.8927 (4)	0.06421 (16)	0.0682 (8)	
H7A	0.0192	0.8263	0.0520	0.102*	
H7B	0.0870	0.9060	0.0188	0.102*	
H7C	0.0437	0.9984	0.0820	0.102*	
C8	0.20235 (11)	0.9857 (3)	0.09561 (13)	0.0423 (5)	
C9	0.34432 (13)	1.0263 (3)	−0.02897 (14)	0.0508 (6)	
C10	0.36834 (13)	1.1545 (3)	−0.08374 (13)	0.0499 (6)	
C11	0.32524 (14)	1.2794 (3)	−0.10794 (15)	0.0644 (7)	
H11	0.2792	1.2825	−0.0921	0.077*	
C12	0.35119 (18)	1.3991 (4)	−0.15583 (18)	0.0836 (10)	
H12	0.3231	1.4851	−0.1723	0.100*	
C13	0.41887 (19)	1.3901 (4)	−0.17897 (18)	0.0831 (10)	
H13	0.4377	1.4695	−0.2114	0.100*	
C14	0.45800 (16)	1.2616 (4)	−0.15322 (16)	0.0735 (9)	
H14	0.5040	1.2561	−0.1690	0.088*	
C15	0.38792 (17)	0.8767 (4)	−0.0182 (2)	0.1043 (13)	
H15A	0.4022	0.8692	0.0349	0.156*	
H15B	0.4281	0.8838	−0.0508	0.156*	
H15C	0.3615	0.7810	−0.0317	0.156*	
H1	0.2784 (13)	0.8410 (17)	0.0589 (16)	0.080*	

### Atomic displacement parameters (Å^2^)

**Table:**

	*U*^11^	*U*^22^	*U*^33^	*U*^12^	*U*^13^	*U*^23^
N1	0.0503 (11)	0.0302 (10)	0.0547 (12)	0.0008 (9)	0.0072 (10)	0.0072 (10)
N2	0.0514 (11)	0.0343 (10)	0.0492 (12)	−0.0062 (9)	0.0018 (10)	0.0042 (9)
N3	0.0678 (14)	0.0579 (14)	0.0611 (14)	−0.0096 (11)	0.0209 (11)	−0.0052 (11)
O1	0.0568 (10)	0.0342 (9)	0.0930 (14)	0.0044 (8)	0.0086 (9)	0.0081 (9)
C1	0.0511 (14)	0.0319 (12)	0.0438 (13)	−0.0001 (10)	0.0069 (11)	−0.0016 (10)
C2	0.0489 (14)	0.0517 (15)	0.0533 (15)	−0.0068 (11)	0.0079 (12)	−0.0033 (12)
C3	0.0606 (17)	0.083 (2)	0.081 (2)	−0.0229 (16)	0.0119 (16)	0.0115 (18)
C4	0.090 (2)	0.078 (2)	0.082 (2)	−0.0174 (18)	0.0242 (19)	0.0276 (18)
C5	0.088 (2)	0.0617 (18)	0.0567 (17)	0.0042 (16)	0.0064 (15)	0.0158 (14)
C6	0.0614 (15)	0.0422 (14)	0.0573 (16)	0.0013 (12)	0.0007 (13)	0.0023 (12)
C7	0.0512 (15)	0.082 (2)	0.0719 (18)	−0.0008 (14)	−0.0049 (14)	0.0016 (16)
C8	0.0439 (13)	0.0300 (12)	0.0531 (14)	−0.0024 (10)	−0.0038 (11)	0.0020 (11)
C9	0.0557 (14)	0.0390 (14)	0.0576 (15)	0.0000 (11)	0.0078 (12)	0.0013 (12)
C10	0.0613 (16)	0.0434 (14)	0.0450 (14)	−0.0095 (12)	0.0070 (12)	−0.0049 (11)
C11	0.0624 (16)	0.0629 (17)	0.0678 (18)	−0.0059 (14)	−0.0004 (14)	0.0220 (15)
C12	0.092 (2)	0.081 (2)	0.078 (2)	−0.0112 (18)	−0.0011 (18)	0.0332 (18)
C13	0.105 (3)	0.081 (2)	0.0635 (19)	−0.032 (2)	0.0128 (18)	0.0146 (17)
C14	0.084 (2)	0.075 (2)	0.0621 (19)	−0.0227 (18)	0.0271 (16)	−0.0054 (17)
C15	0.090 (2)	0.076 (2)	0.147 (3)	0.0296 (18)	0.052 (2)	0.046 (2)

### Geometric parameters (Å, °)

**Table:**

N1—C8	1.354 (3)	C6—H6	0.9300
N1—N2	1.384 (2)	C7—H7A	0.9600
N1—H1	0.907 (10)	C7—H7B	0.9600
N2—C9	1.280 (3)	C7—H7C	0.9600
N3—C14	1.332 (3)	C9—C10	1.484 (3)
N3—C10	1.338 (3)	C9—C15	1.492 (3)
O1—C8	1.225 (2)	C10—C11	1.379 (3)
C1—C6	1.386 (3)	C11—C12	1.374 (4)
C1—C2	1.400 (3)	C11—H11	0.9300
C1—C8	1.490 (3)	C12—C13	1.368 (4)
C2—C3	1.383 (3)	C12—H12	0.9300
C2—C7	1.501 (3)	C13—C14	1.365 (4)
C3—C4	1.369 (4)	C13—H13	0.9300
C3—H3	0.9300	C14—H14	0.9300
C4—C5	1.365 (4)	C15—H15A	0.9600
C4—H4	0.9300	C15—H15B	0.9600
C5—C6	1.379 (3)	C15—H15C	0.9600
C5—H5	0.9300		
			
C8—N1—N2	117.17 (17)	H7B—C7—H7C	109.5
C8—N1—H1	121.7 (17)	O1—C8—N1	123.0 (2)
N2—N1—H1	120.7 (18)	O1—C8—C1	121.2 (2)
C9—N2—N1	118.60 (19)	N1—C8—C1	115.71 (19)
C14—N3—C10	117.3 (2)	N2—C9—C10	114.9 (2)
C6—C1—C2	120.6 (2)	N2—C9—C15	126.5 (2)
C6—C1—C8	120.8 (2)	C10—C9—C15	118.5 (2)
C2—C1—C8	118.7 (2)	N3—C10—C11	122.2 (2)
C3—C2—C1	117.1 (2)	N3—C10—C9	116.2 (2)
C3—C2—C7	120.4 (2)	C11—C10—C9	121.6 (2)
C1—C2—C7	122.5 (2)	C12—C11—C10	119.1 (3)
C4—C3—C2	122.3 (3)	C12—C11—H11	120.4
C4—C3—H3	118.8	C10—C11—H11	120.4
C2—C3—H3	118.8	C13—C12—C11	119.1 (3)
C5—C4—C3	120.1 (3)	C13—C12—H12	120.4
C5—C4—H4	120.0	C11—C12—H12	120.4
C3—C4—H4	120.0	C14—C13—C12	118.3 (3)
C4—C5—C6	119.7 (3)	C14—C13—H13	120.9
C4—C5—H5	120.1	C12—C13—H13	120.9
C6—C5—H5	120.1	N3—C14—C13	124.0 (3)
C5—C6—C1	120.2 (2)	N3—C14—H14	118.0
C5—C6—H6	119.9	C13—C14—H14	118.0
C1—C6—H6	119.9	C9—C15—H15A	109.5
C2—C7—H7A	109.5	C9—C15—H15B	109.5
C2—C7—H7B	109.5	H15A—C15—H15B	109.5
H7A—C7—H7B	109.5	C9—C15—H15C	109.5
C2—C7—H7C	109.5	H15A—C15—H15C	109.5
H7A—C7—H7C	109.5	H15B—C15—H15C	109.5

### Hydrogen-bond geometry (Å, °)

**Table:**

*D*—H···*A*	*D*—H	H···*A*	*D*···*A*	*D*—H···*A*
N1—H1···O1^i^	0.91 (1)	2.05 (1)	2.937 (2)	165 (3)

Symmetry codes: (i) −*x*+1/2, *y*−1/2, *z*.
